# Resting state EEG oscillatory power differences in ADHD college students and their peers

**DOI:** 10.1186/1744-9081-8-60

**Published:** 2012-12-18

**Authors:** Steven Woltering, Jessica Jung, Zhongxu Liu, Rosemary Tannock

**Affiliations:** 1 Applied Psychology and Human Development, Ontario Institute for Studies in Education, University of Toronto, Toronto, Canada; 2 Neuroscience and Mental Health Research Program, Hospital for Sick Children, Toronto, Ontario, Canada

**Keywords:** Quantitative Electroencephalography (EEG), Adults, Power, Attention-deficit/hyperactivity disorder (ADHD), Resting state, Alpha, Beta, Theta, Intra-individual variability, Eyes open, Eyes closed

## Abstract

**Background:**

Among the most robust neural abnormalities differentiating individuals with Attention-Deficit/Hyperactivity Disorder (ADHD) from typically developing controls are elevated levels of slow oscillatory activity (e.g., theta) and reduced fast oscillatory activity (e.g., alpha and beta) during resting-state electroencephalography (EEG). However, studies of resting state EEG in adults with ADHD are scarce and yield inconsistent findings.

**Methods:**

EEG profiles, recorded during a resting-state with eyes-open and eyes-closed conditions, were compared for college students with ADHD (n = 18) and a nonclinical comparison group (n = 17).

**Results:**

The ADHD group showed decreased power for fast frequencies, especially alpha. This group also showed increased power in the slow frequency bands, however, these effects were strongest using relative power computations. Furthermore, the theta/beta ratio measure was reliably higher for the ADHD group. All effects were more pronounced for the eyes-closed compared to the eyes-open condition. Measures of intra-individual variability suggested that brains of the ADHD group were less variable than those of controls.

**Conclusions:**

The findings of this pilot study reveal that college students with ADHD show a distinct neural pattern during resting state, suggesting that oscillatory power, especially alpha, is a useful index for reflecting differences in neural communication of ADHD in early adulthood.

## Background

Attention-deficit/hyperactivity disorder (ADHD) is a pervasive mental health condition that is characterized by symptoms of inattention and hyperactivity. About 5% of children are estimated to meet the diagnosis of ADHD worldwide [1]. Adults with ADHD, particularly the subset that pursue post-secondary education, are an understudied population despite research showing that over 50% of children with ADHD continue to show symptoms in adulthood [2-4]. ADHD adults often show a decrease in their hyperactivity symptoms, but symptoms relating to cognitive impairments, although less marked, remain [5-7]. Despite an apparent age-related decline in symptoms, problems with inattention, working memory, and an increased mental restlessness continue to undermine their occupational/academic functioning and raise their risk for psychopathology (e.g., depression, anxiety), and substance abuse [5,8-10].These problems present unique challenges to individuals in post-secondary educational settings that demand self-discipline and higher order executive functioning such as attentional control.

In the last few years, research investigating ADHD populations has used neurophysiological measures such as EEG oscillatory power to determine whether ADHD can be distinguished by specific neural abnormalities [11]. Neuronal oscillations are an important mechanism enabling coordinated communication in a neural network [12]. Different neural oscillations observed during a resting state represent brain activity at different spatial and temporal scales, and these cortical oscillation profiles may underlie particular ADHD symptomatology. For example, childhood ADHD has been characterized by higher power in slow oscillations (e.g., delta and theta frequencies), and lower power in fast oscillations (e.g., alpha and beta frequencies) relative to normative control groups (see, [13,14], for reviews). A ratio measure, dividing slow-oscillatory by fast-oscillatory power, has shown to be one of the most reliable neurophysiological indices of ADHD [14], although its reliability for diagnoses remains uncertain [15].

However, the specificity of this EEG power profile to adult ADHD, and its meaning, are under debate [16,17]. Similar abnormalities in EEG power have been observed in patients with head injuries, dementia, and schizophrenia [16,18,19], indicating a more general atypical neural functioning or organization. The EEG profile seen with ADHD has been interpreted in various ways: the relatively low power in fast oscillations is consistent with the cortical hypo-arousal theory giving rise to reduced executive functioning and self-control [20], whereas the high power in slow oscillations could reflect diminished control of strong subcortical drives and impulses [21]. Another perspective builds on work showing that, compared to normally developing children, children with ADHD display a neural oscillatory pattern that resembles younger children [22], providing support for the maturational lag model of ADHD which explains their symptoms as being developmentally inappropriate [23].

Compared to studies of children with ADHD, research examining oscillatory power in adults with ADHD has been scarce and findings have been inconsistent. For example, in keeping with the child ADHD literature, a higher power in slow oscillations has been found for ADHD adults compared to healthy comparison groups in some studies [24,25], but this finding has not been replicated in another [26]. The discrepancies become more complex when examining fast oscillations. Consistent with the child ADHD literature, Bresnahan et al., [24] found ADHD adults to have lower beta power. However, this finding was only valid for *relative* beta power (Indicates the power of a specific band relative to power in all bands) because no group differences were found in the beta and alpha bands for absolute power. In contrast, Clarke et al., [26] found higher absolute and relative beta power for ADHD groups, and Koehler et al., [25] found that ADHD adults have higher absolute alpha power. It is possible methodological differences might account for some of the inconsistencies. For example, some studies measured the resting state EEG during an eyes-closed condition [25,26], whereas others recorded during an eyes-open condition [24,27]. No strong theoretical framework for interpreting differences for both eye-conditions exists in the literature, partly because very few studies have explicitly examined differences between both conditions in ADHD. The most consistent finding remains an elevated theta/beta ratio, however, only a few studies so far have examined this in adults [24,25,27,28].

The present pilot study investigated neural oscillatory power during a resting state in college students with ADHD and their normal healthy peers. To the best of our knowledge, this is the first study to investigate oscillatory power in college students with ADHD. This relatively successful subset, accepted into post-secondary education, continues to manifest cognitive and other functional impairments [9,11]. In addition to computing measures of absolute power and relative power, we also examined eyes-open and eyes-closed conditions as differences in these measures might explain discrepancies seen in the literature. Furthermore, we also explored measures of intra-individual variability in brain activation.

## Methods

### Participants

Eighteen participants with ADHD (8 male; 1 left-handed; mean age = 25.8, *sd* = 4.27) were recruited from University Student Services and 17 normal healthy controls were recruited through campus advertisements (10 male; 2 left-handed; mean age = 24.4, *sd* = 4.39). Inclusion criteria were 1) current enrollment in a post-secondary program, 2) a previous diagnosis of ADHD, and 3) registration with respective university or college Student Disability Services, which requires supporting documentation of a confirmed diagnosis of ADHD. All participants completed the Adult ADHD Self Report Scale (ASRS) to assess current symptoms of ADHD. Exclusion criteria were 1) uncorrected sensory impairment, 2) major neurological dysfunction and psychosis, and 3) current use of sedating or mood altering medication other than stimulants prescribed for ADHD. Among the clinical sample, 10 subjects (56%) were being treated with medication. Of those 10 subjects, 6 subjects were using stimulants only, 2 subjects were using a combination of stimulants and antidepressants, and 2 subjects were using a combination of stimulants, antidepressants, and other non-prescriptive medications. Participants were asked not to change their medication treatment when visiting the lab for assessment. Three participants had a comorbid learning disability, and one participant was diagnosed with anxiety and depression.

### Procedure

The present study was approved by the University of Toronto Research Ethics Board (protocol reference #23977) and all participants provided informed written consent prior to the start of the study.

Participants were seated in a comfortable chair and fitted with a 129-channel EEG net (Electrical Geodesic Inc., EGI). Acquisition started after impedances for all channels were reduced to below 50 kΩ in accordance with standard data collection procedures [29,30]. Data were collected using a . 1 – 1000 Hz bandpass hardware filter and a 500 Hz sampling rate. Data were referenced to electrode Cz. After becoming familiar with the environment, instructions on a screen explained the task to the participants. A sound signaled when they were to alternate closing or opening their eyes. Participants did this for six 40 second intervals (i.e., 120 seconds for each condition). Participants were encouraged to relax, prevent excessive blinking, and to keep the eyes fixated on a central cross to prevent eye-movements during eyes-open.

### Behavioural measures

Each participant completed a number of standard questionnaires and tasks to assess current symptom impairment:

The Adult ADHD Self-Report Scale (ASRS v1.1) is a reliable and valid scale for evaluating current ADHD symptoms in adults [31]. The ASRS v1.1 consists of eighteen questions based on the criteria used for diagnosing ADHD in the DSM-IV-TR. Scores for each item were added to calculate a total score. Subtypes were not investigated considering recent conclusions drawn in the literature questioning their validity [32].

The Cognitive Failures Questionnaire (CFQ) measures self-reported failures in perception, memory, and motor function in everyday life. Twenty-five questions ask subjects to rank how often these mistakes occur [33].

The Reading Fluency subscale of the Woodcock Johnson-III Tests of Achievement [34] was administered to determine automaticity of identifying words, to provide a confirmatory index of Specific Learning Disabilities. The dependent variable was the number of correctly completed items in three minutes. All subtest raw scores were converted to standard scores.

The Digit Span subtest from the Wechsler Adult Intelligence Scale- Fourth Edition (WAIS-IV) was used to assess auditory-verbal working memory, as a crude index of executive function [35]. The Digit Span raw score was converted to an age-adjusted scaled score.

### EEG data processing

Netstation (Electrical Geodesic Inc, EGI) was used to filter (FIR, .1-100 Hz, excluding 60 Hz notch) and segment the data into 2-second segments (e.g., 60 segments per condition). Segments containing artifacts were removed using standard, automatic algorithms for the detection of eye blinks, eye movements, as well as large drifts, and spikes in the data. Segments containing more than 20% bad channels were automatically removed. In addition, all segments were visually inspected by a trained research assistant blind to the hypotheses. Bad channels were replaced by values interpolated from neighboring channel data. Across all subjects, an average of 1 channel needed to be replaced. The ADHD group had an average of 38 useful segments (*sd* = 13.6) in the eyes-closed condition and 43 segments (*sd* = 12.7) during eyes-open, whereas the control group had an average of 43 segments (*sd* = 14.2) in the eyes-closed condition and 46 segments *(sd* = 9.5) during eyes-open. Groups did not significantly differ in segment count (*p*’s > .25). No subjects were discarded for meeting our cutoff criterion of less than 11 segments.

Next, data were exported to MATLAB 7.5 (The Mathworks, Inc.) for further analysis.

In MATLAB, the data were average referenced, and a Fast Fourier Transform was run using the pwelch algorithm, with a 128 sample triangular window, to obtain time-frequency domain measures. Mean spectral estimates in various power bands (theta, 4–7; alpha, 8–12; beta, 13–25) were computed. As a measure of intra-individual variability in neural activity, the standard deviation of the power in each 2-second trial was calculated for each band for each participant. To reduce unnecessary computation and multiple tests, we chose to extract data for 66 channels based on the standard EGI template.

### Statistical analysis

To examine potential outliers, the modified thompson-tau method was used. In addition to absolute power, the relative power of each frequency band (the amount of power in one band divided by the power in all other bands) was also computed for the eyes-open as well as eyes-closed conditions to permit comparison with other studies. Independent sample t-tests were used to assess differences at each electrode between the ADHD and control groups. Data were presented in difference plots showing the t-value test statistic between groups at each electrode. Significant effects are indicated on the plots using dots at a .05 level, and using crosses when significance was reached using the conservative Bonferroni correction for multiple comparisons. This manner of presenting data instantly shows the direction as well as the significance of the differences found between groups, and provides an overview of the spatial variability of the data across the scalp.

## Results

### Group characteristics

The ASRS confirmed that the ADHD group, relative to the control group, exhibited more ADHD symptoms (*p*’s < .001). Furthermore, the CFQ showed that the ADHD group reported significantly more general cognitive failures in their everyday life (all *p’*s *<* .001).

Both groups had a comparable number of years of education, and performed similarly on tests of reading fluency and working memory, and also didn’t differ in age. Table 1 shows the means and standard deviations for the ADHD and control group for each of the questionnaires and tasks.

**Table 1:** Questionnaire and task results for the ADHD and control group

	**Clinical (n = 18)*(mean/sd)***	**Control (n = 17)*(mean/sd)***
**Years of Education**	15.7 (1.2)	15.9 (1.7)
ASRS^***^	51.4 (11.7)	20.0 (12.7)
CFQ^***^	57.3 (11.2)	26.8 (10.1)
**WJ-III Reading fluency**	97.9 (14.5)	104.7 (18.4)
**WAIS - Digit Span**	8.3 (3.4)	9.4 (2.7)

Independent sample t-tests between clinical and control group, * p < .05, ** p < .01, *** p < .001.

ASRS, Adult ADHD Self Report Scale; CFQ, Cognitive Failures Questionnaire; WJ-III, Woodcock Johnson III Tests of Achievement; WAIS, Wechsler Adult Intelligence Scale.

Participants on medication (*m* = 55.80, *sd* = 12.56) reported more ADHD symptoms on the ASRS, at a trend level, than those who were not using medication (*m* = 45.88, *sd* = 8.06), *t*(16) = 1.93, *p* = .07. It is possible that those subjects who were not using medication had less severe symptoms to begin with.

### Absolute power

**Figure 1:**
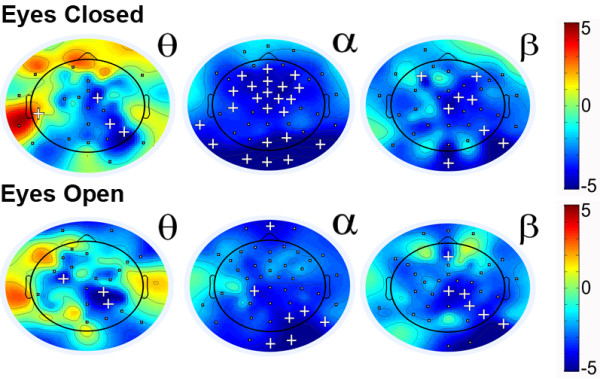
**t-scores for differences in absolute power between ADHD and control group across the scalp for the eyes-closed (top) and eyes-open (bottom) condition for the theta (θ), alpha (α), and beta (β) bands.** Greater values (red) represent higher power in the ADHD group. Squares (▪) indicate significance at *p* < .05, and plus signs (**+**) indicate significance *p* < .0008 (corrected for multiple comparisons - Bonferroni).

For the eyes-closed condition, significant differences using t-tests (*p’*s < .05) between groups were found for slow as well as fast oscillations. Slow oscillations (theta) showed increased power for ADHD compared to the control group for anterior and lateral electrodes. However, *lower* power in slow oscillations was found in more central and posterior electrodes. As expected, the ADHD group exhibited lower power in the fast oscillations for all electrodes. This pattern of results also held when investigating those subjects with ADHD who used medication. A similar pattern of differences was found for the eyes-open condition, however, the increases in slow oscillations seemed less pronounced. Figure 1 shows the scalp difference plots between the groups for each power band for the eyes-closed as well as eyes-open condition.

To test whether the two Eye conditions (eyes-open, eyes-closed) differed between ADHD and controls, a 2 (Group) x 2 (Eye Condition) Mixed Model ANOVA looking at electrode sites Fz, Cz, Pz, and Oz was conducted with medication as a covariate. A main effect of Eye condition was found in the alpha band for all electrodes (*p*’s < .05) with, as expected, the eyes-closed condition showing increased alpha compared to eyes-open. A significant main effect of Eye Condition was also found for the Beta band, but only for electrode site Pz (*p* < .05). Significant Group by Eye Condition interactions were found for power in the alpha band at electrode site Fz, *F*(1, 32) = 7.83, *p* < .01, as well as Oz, *F*(1,32) = 4.63, *p* < .05. Post-hoc analyses showed that the comparison group had a stronger decrease in alpha power compared to the ADHD group from eyes-closed to eyes-open. This is partly due to the comparison group having higher alpha power during the eyes-closed condition.

Group differences in intra-individual variability in neural oscillatory power across the different frequency bands were tested for electrode sites Fz, Cz, Pz, and Oz. The ADHD group showed significantly less variability in the fast oscillatory band as shown by multiple t-tests, in particular for the central electrode for alpha and beta, and the occipital electrodes for alpha (p’s < .0008, corrected for multiple comparisons). Mean, sample standard deviation, and intra-individual standard deviation values are presented in additional file 1 for each band and for each electrode site for eyes-closed (Additional file 1: Table A1) as well as eyes-open (Additional file 1: Table A2).

### Relative power

**Figure 2:**
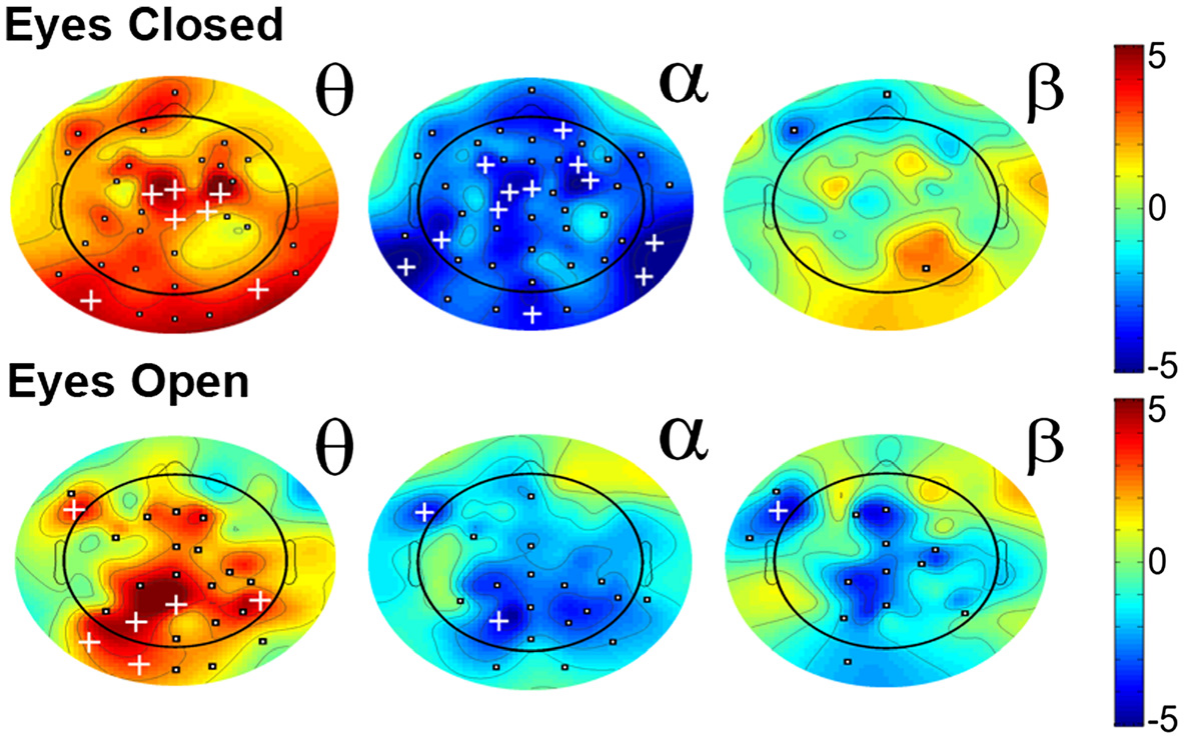
**t-scores for differences in relative power between ADHD and control group for the eyes-closed (top) and eyes-open (bottom) condition for the theta (θ), alpha (α), and beta (β) bands.** Greater values (red) represent higher power in the ADHD group. Squares (▪) indicate significance at *p* < .05, and plus signs (**+**) indicate significance *p* < .0008 (corrected for multiple comparisons - Bonferroni).

For the eyes-closed condition, the ADHD group showed significantly higher relative power in the slow oscillatory bands compared to the control group. This effect was present across the entire scalp. Fast oscillations showed significantly smaller power for the ADHD compared to the control group. These effects were most pronounced in the alpha band. We note that a similar pattern of results was present for just those ADHD on medication. The eyes-open condition also showed a similar pattern, however, the effects were not as strong. Figure 2 shows the differences between the groups in scalp plots for each condition and each power band (values of Fz, Cz, Pz, and Oz for each eye condition are presented in Additional file 1, Table A1 and A2).

**Figure 3:**
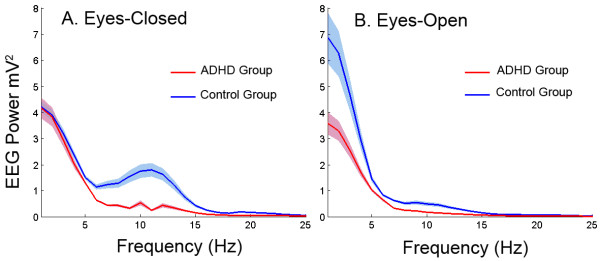
**Power (mV**^**2**^**) by frequency (Hz) plot at Electrode Cz for the ADHD (red) and the control (blue) groups in the eyes-closed (A) and eyes-open condition (B).** Theta, 4–7 Hz; alpha, 8–12 Hz; beta, 13–25 Hz. Shaded area represents standard error.

To illustrate the contributions of various bands underlying the relative power differences, a power-by-frequency plot (see Figure 3) was computed at electrode site Cz only for the eyes-closed and eyes-open condition. The ADHD group showed decreased power compared to the control group in the alpha (*p* < .0001) and beta (*p* < .001) band. We note that the strong decrease in alpha power for the ADHD group is indirectly driving the *relatively* larger contribution of slow wave oscillations shown in computations of relative power.

**Figure 4:**
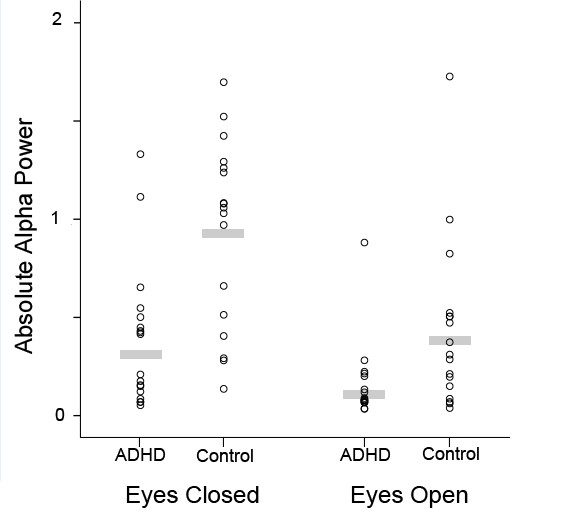
**Individual data points for Absolute power (mV**^**2**^**) at electrode site Cz for the ADHD and control groups for the eyes-closed and eyes-open conditions.**

Figure 4 shows the data points for each group for absolute power in the alpha band at electrode Cz.

### Ratio power values

**Figure 5:**
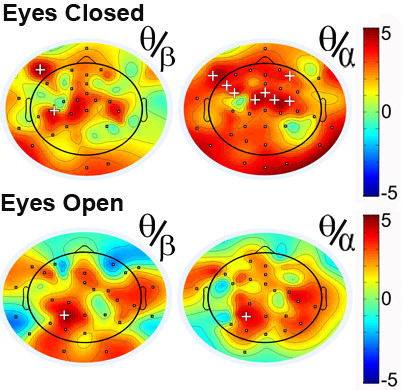
**t-scores for differences in ratio power between ADHD and control group for the eyes-closed (top) and eyes-open (bottom) condition for the Theta/beta Ratio (θ/β, left) and Theta/alpha ratio (θ/α, right).** Greater values (red) represent higher power in the ADHD group. Squares (▪) indicate significance at *p* < .05, and plus signs (**+**) indicate significance *p* < .0008 (corrected for multiple comparisons - Bonferroni).

For the eyes-closed condition, the theta/beta ratio was significantly higher in the ADHD group compared to the control group at anterior and lateral electrode sites. A theta/alpha ratio yielded a similar but stronger pattern, for the entire scalp. Results for the eyes-open condition showed a similar pattern, however, effects were generally weaker (see, Figure 5).

## Discussion

In the present study, young adults with ADHD showed decreased oscillatory power in fast frequencies, particularly within the alpha band, relative to normal healthy controls. Increased power for slow frequencies was also found for individuals with ADHD, although these results were more specific to measures of relative power. The ADHD group showed a higher theta/beta ratio compared to the control group in fronto-central and lateral electrode sites, confirming the ratio measure to be associated with ADHD, even in adulthood. Generally, these results replicate the findings found in childhood ADHD that show increased slow oscillatory power and decreased fast oscillatory power [13,14]. Because variables such as age, sex, and estimates of executive function and years of education were similar between groups, different oscillatory activation patterns likely represent differences in neural communication related to ADHD symptomatology.

Our findings are consistent with some studies done on adults pertaining to higher power in slow oscillations [27,36], lower power in fast oscillations [37] and an higher ratio values [24,25,27,28]. But there are discrepancies with other studies, in particular results reporting increases in fast oscillatory power bands [25,26]. More studies are required to investigate the nature of these discrepancies in the high frequency oscillations. One possible explanation for the discrepancies might be the nature of our sample that consists out of relatively high-functioning college students.

The differences found in the current study may reflect a different neurophysiology in this specific ADHD population of young adults. During resting state, different frequencies of neural oscillations coexist and may interact with each other to maintain a physiological and functional balance in the brain. Low frequency oscillations (e.g., theta) can coordinate long-distance brain regions and function in larger temporal scales [12]. Furthermore, low frequencies oscillations are more prominent in deep cortical laminar regions, which are more easily influenced by cortical-subcortical interaction [38]. Instead, high frequency oscillations (e.g., alpha, beta) measured by scalp EEG may reflect more local cortical computation for executive, memory, and motor functions [39,40]. The increased power in slow oscillations, and especially the reduced fast oscillatory power, may reflect an unbalanced or non-optimal interaction among local cortical neural activities and long-range corticocortical/cortico-subcortical neural activities, which may be related to their ADHD symptomatology.

Although the child ADHD literature mostly focuses on power in theta and beta bands [13,14], it seems the results in our study of adult students are mostly driven by differences in alpha power. First, the strongest, most and widespread, differences between the ADHD and control group are seen in the alpha band. Second, the effects of alpha were the most reliable, as they held for computations of absolute as well as relative power, and for the eyes-closed as well as the eyes-open condition. Third, the group differences found for *relative* power in the slow frequency bands can be attributed to decreases in alpha. Last, the theta/alpha ratio showed even stronger group differences than the theta/beta ratio.

We suggest that the lower alpha seen in the ADHD group may be related to problems in attentional self-control. Recently, alpha power has been associated with active inhibition of external stimuli in a variety of tasks [41]. This framework would suggest that more alpha desynchronization may reflect an increased focus on the processing of external stimuli. However, subjects in a non-task related, relaxing state generally do not actively process external stimuli to great extent. It is possible that the neural circuitry of people with ADHD is wired such that they are more attuned to process external stimuli, and that the decreased alpha power is a reflection of this propensity. Such increased vigilance to external stimuli could be beneficial in certain contexts, however, when attention needs to be consistently directed to internal goals, it may become problematic. Though speculative, this interpretation could complement Rowe et al.’s [20] account that individuals with ADHD suffer from a lack of inhibition over sensory input, and might explain the distractibility and concentration problems adults with ADHD experience, and specifically the student population.

The current study also investigated differences in oscillatory power between the eyes-open and eyes-closed conditions as well as the intra-individual variability of that power. Concerning eyes-open and eyes-closed conditions, based on our data we conclude that both conditions are relatively similar between groups, however, the effects of higher slow oscillatory power and lower fast oscillatory power seemed more pronounced during the eyes-closed condition for alpha in the ADHD group. It is possible that the eyes-closed condition is a better, or cleaner, reflection of intrinsic alpha oscillation because visual input from the thalamus during the eyes-open condition may 'disturb' alpha rhythms mediated by cortico-thalamic loops [42,43]. Concerning intra-individual variability, interestingly, the ADHD group showed *less* inter-trial variability in power, particularly in fast oscillations such as alpha. These results agree with emerging notions in the field suggesting low variability of metastable brain states are associated with less behavioural stability [44,45]. Indeed, one characteristic of people with ADHD is a high variability in task performance [46]. We shall further explore this phenomenon in future studies.

A limitation of this study is its relatively low sample size, which constrained our ability to investigate the effects of comorbidities. Furthermore, this study included subjects who were on medication. Questionnaire data indicated that ADHD symptomatology was stronger among those taking medication compared to those in the ADHD group, however, analyses demonstrated that subjects on medication showed a similar pattern of differences with the control group for absolute as well as relative power. Furthermore, we point out that previous studies have found that stimulants tend to normalize EEG oscillatory power on ADHD adults [24], suggesting effects would have been stronger had we excluded those subjects on medication.

## Conclusions

These data suggest that the neurophysiological differences found between individuals with ADHD and their peers in childhood are also present in adulthood. The findings may help document the behavioral and neural nature of adult ADHD, which may eventually lead to a better understanding and treatment.

## Abbreviations

ADHD: Attention-Deficit/Hyperactivity Disorder; EEG: Electroencephalography; ASRS: Adult ADHD Self Report Scale; CFQ: Cognitive Failures Questionnaire; WJ-III: Woodcock Johnson-III Tests of Achievement; WAIS-IV: Wechsler Adult Intelligence Scale- Fourth Edition.

## Competing interests

The authors declare that they have no competing interests.

## Authors’ contributions

SW, ZL and RT contributed to the conception and design of the study. SW and JJ were responsible for data acquisition, performed the data processing and analyses, and drafted the manuscript. ZL assisted with the power analyses. RT and ZL helped critically revise the manuscript. All authors read and approved the final manuscript.

## Supplementary Material

Additional file 1 **Table A1.** Mean (sample standard deviation, inter-trial standard deviation) in absolute (mV^2^) and relative power for theta, alpha, and beta bands between the ADHD and control group for electrodes Fz, Cz, Pz, and Oz in the eyes-closed condition. **Table A2.** Mean (sample standard deviation) in absolute (mV^2^) and relative power for theta, alpha, and beta bands between the ADHD and control group for electrodes Fz, Cz, Pz, and Oz in the eyes-open condition. **Figure A3.** Variability in absolute Alpha power (in mV^2^) for electrode 40 in the eyes closed condition for ADHD subjects on, or off, medication. **Figure A4.** Power (in mV^2^) x Frequency plot for eyes-closed for Controls, ADHD participants who were on medication, and those ADHD participants who were not. Click here for file
