# Supplementary material for: Resting state EEG oscillatory power differences in ADHD college students and their peers
Source: Behav Brain Funct. 2012 Dec 18;8:60. doi: 10.1186/1744-9081-8-60 (PMC3537601; doi:10.1186/1744-9081-8-60)

**ADDITIONAL FILE 1**

**Table A1**: Mean (sample standard deviation, inter-trial standard deviation) in absolute (mV^2^) and relative power for theta, alpha, and beta bands between the ADHD and control group for electrodes Fz, Cz, Pz, and Oz in the eyes-closed condition.

| Absolute Power | ADHD |  |  | Control |  |  |
| --- | --- | --- | --- | --- | --- | --- |
|  | **Theta** | **Alpha** | **Beta** | **Theta** | **Alpha** | **Beta** |
| Fz | **4.16  (3.46, 5.78)** | **0.92 (0.66, 0.45)** | **0.21 (0.10, 0.09)** | **6.17  (6.01, 13.14)** | **1.88 (1.18, 0.92)** | **0.35  (0.19, 0.16)** |
| Cz | **1.20  (0.90, 0.89)** | **0.77  (0.69, 0.39)** | **0.16  (0.10, 0.07)** | **2.93  (4.04, 12.85)** | **1.76  (1.04, 0.94)** | **0.36  (0.23, 0.19)** |
| Pz | **1.64  (0.90, 1.77)** | **1.58  (1.77, 0.89)** | **0.35  (0.27, 0.18)** | **2.60  (2.14, 2.80)** | **2.74  (2.23, 1.47)** | **0.55  (0.37, 0.27)** |
| Oz | **2.08  (1.47, 2.49)** | **1.84 (2.23, 0.86)** | **0.43  (0.35, 0.19)** | **2.38  (1.49, 2.82)** | **3.57 (2.67, 1.82)** | **0.72  (0.43, 0.34)** |

| **Relative Power X 10^-2^** | **ADHD** | |  | |  | | **Control** | |  | |  | |  |  |
| --- | --- | --- | --- | --- | --- | --- | --- | --- | --- | --- | --- | --- | --- | --- |
|  | | **Theta** | | **Alpha** | | **Beta** | | **Theta** | | **Alpha** | | **Beta** | | |
| **Fz** | | **20.18 (3.53)** | | **2.49 (2.01)** | | **0.52 (0.34)** | | **18.70 (4.41)** | | **3.56 (2.60)** | | **0.57 (0.40)** | | |
| **Cz** | | **14.27 (4.75)** | | **5.50 (3.11)** | | **1.19 (0.50)** | | **12.93 (6.59)** | | **6.66 (4.20)** | | **1.15 (0.68)** | | |
| **Pz** | | **12.90 (6.18)** | | **6.04 (3.83)** | | **1.40 (0.62)** | | **11.16 (6.13)** | | **7.37 (3.67)** | | **1.43 (0.92)** | | |
| **Oz** | | **13.96 (6.57)** | | **5.23 (3.81)** | | **1.39 (0.79)** | | **9.69 (4.47)** | | **8.26 (2.95)** | | **1.53 (0.73)** | | |

**Table A2**: Mean (sample standard deviation) in absolute (mV^2^) and relative power for theta, alpha, and beta bands between the ADHD and control group for electrodes Fz, Cz, Pz, and Oz in the eyes-open condition.

| Absolute Power | ADHD |  |  | Control |  |  |
| --- | --- | --- | --- | --- | --- | --- |
|  | **Theta** | **Alpha** | **Beta** | **Theta** | **Alpha** | **Beta** |
| Fz | **2.27 (3.16)** | **0.18 (0.16)** | **0.05 (0.02)** | **3.57 (4.44)** | **0.30 (0.24)** | **0.08 (0.05)** |
| Cz | **2.09 (3.75)** | **0.16 (0.19)** | **0.04 (0.02)** | **2.26 (2.03)** | **0.43 (0.43)** | **0.10 (0.11)** |
| Pz | **2.35 (3.63)** | **0.30 (0.44)** | **0.07 (0.07)** | **1.70 (2.04)** | **0.60 (0.50)** | **0.12 (0.10)** |
| Oz | **2.27 (2.65)** | **0.28 (0.35)** | **0.08 (0.06)** | **1.49 (1.79)** | **0.53 (0.64)** | **0.12 (0.09)** |

| Relative  Power X 10^-2^ | ADHD |  |  | Control |  |  |
| --- | --- | --- | --- | --- | --- | --- |
|  | **Theta** | **Alpha** | **Beta** | **Theta** | **Alpha** | **Beta** |
| Fz | **18.86 (4.66)** | **2.78 (2.42)** | **0.82 (0.61)** | **18.35 (4.65)** | **3.23 (2.73)** | **0.80 (0.51)** |
| Cz | **18.31(5.35)** | **3.30 (3.09)** | **0.79 (0.54)** | **16.17 (5.76)** | **4.57 (3.66)** | **0.96 (0.55)** |
| Pz | **17.42 (5.29)** | **3.54 (2.91)** | **0.97 (0.59)** | **14.33 (6.19)** | **5.55 (3.65)** | **1.15 (0.72)** |
| Oz | **16.79 (5.65)** | **3.61(3.03)** | **1.14 (0.69)** | **13.77 (6.01)** | **5.48 (3.68)** | **1.35 (0.67)** |

**Figure A3:** Variability in absolute Alpha power (in mV^2^) for electrode 40 in the eyes closed condition for ADHD subjects on, or off, medication.


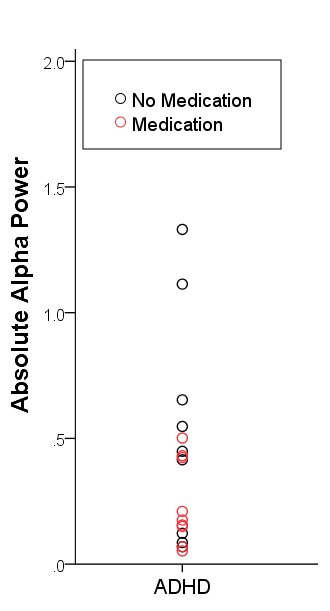


**Figure A4:** Power (in mV^2^) x Frequency plot for eyes-closed for Controls, ADHD participants who were on medication, and those ADHD participants who were not.


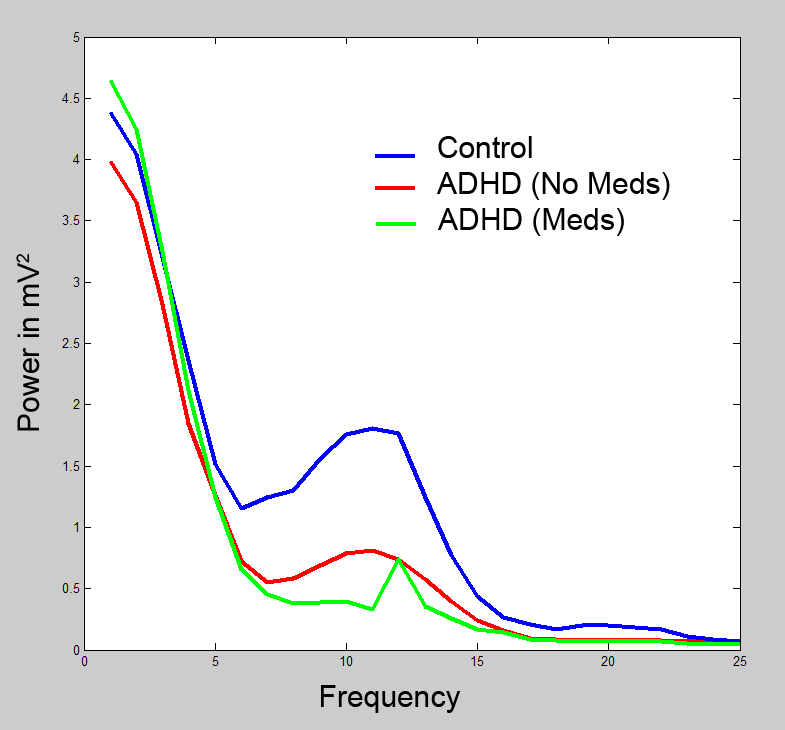

Supplement: Additional file 1 — Table A1. Mean (sample standard deviation, inter-trial standard deviation) in absolute (mV2) and relative power for theta, alpha, and beta bands between the ADHD and control group for electrodes Fz, Cz, Pz, and Oz in the eyes-closed condition. Table A2. Mean (sample standard deviation) in absolute (mV2) and relative power for theta, alpha, and beta bands between the ADHD and control group for electrodes Fz, Cz, Pz, and Oz in the eyes-open condition. Figure A3. Variability in absolute Alpha power (in mV2) for electrode 40 in the eyes closed condition for ADHD subjects on, or off, medication. Figure A4. Power (in mV2) x Frequency plot for eyes-closed for Controls, ADHD participants who were on medication, and those ADHD participants who were not. [file 1744-9081-8-60-S1.docx]
